# A personalized, intense physical rehabilitation program improves walking in people with multiple sclerosis presenting with different levels of disability: a retrospective cohort

**DOI:** 10.1186/s12883-015-0281-9

**Published:** 2015-03-04

**Authors:** Alon Kalron, Dalia Nitzani, David Magalashvili, Mark Dolev, Shay Menascu, Yael Stern, Uri Rosenblum, Diana Pasitselsky, Lior Frid, Gabi Zeilig, Caroline Barmatz, Uri Givon, Anat Achiron

**Affiliations:** Multiple Sclerosis Center, Sheba Medical Center, Tel Hashomer, Israel; Department of Physical Therapy, Sackler Faculty of Medicine, Tel-Aviv University, Tel-Aviv, Israel

**Keywords:** Multiple Sclerosis, Gait, Rehabilitation, Walking, Exercise, Hydrotherapy, Neurological Rehabilitation

## Abstract

**Background:**

People with multiple sclerosis (PwMS) endure walking limitations. To address this restriction, various physical rehabilitation programs have been implemented with no consensus regarding their efficacy. Our objective was to report on the efficacy of an integrated tailored physical rehabilitation program on walking in people with multiple sclerosis categorized according to their level of neurological disability.

**Methods:**

Retrospective data were examined and analyzed. Specifically, data obtained from all patients who participated in the Multiple Sclerosis Center’s 3 week rehabilitation program were extracted for in depth exploration. The personalized rehabilitation program included three major components modified according to the patient’s specific impairments and functional needs: (a) goal directed physical therapy (b) moderately intense aerobic exercise training on a bicycle ergometer and (c) aquatic therapy chiefly oriented to body structures appropriate to movement. Gait outcome measurements included the 10 meter, 20 meter, Timed up and go and 2 minute walking tests measured pre and post the rehabilitation program. Three hundred and twelve people with relapsing-remitting multiple sclerosis were included in the final analysis. Patients were categorized into mild (n = 87), moderate (n = 104) and severely (n = 121) disabled groups.

**Results:**

All clinical walking outcome measurements demonstrated statistically significant improvements, however, only an increase in the 2 minute walking test was above the minimal clinical difference value. The moderate and severe groups considerably improved compared to the mild gait disability group. Mean change scores (%) of the pre-post intervention period of the 2 minute walking test were 19.0 (S.E. = 3.4) in the moderate group, 16.2 (S.E. = 5.4) in the severe group and 10.9 (S.E. = 2.3) in the mild gait disability group.

**Conclusions:**

We presented comprehensive evidence verifying the effects of an intense goal-directed physical rehabilitation program on ambulation in people with multiple sclerosis presenting with different neurological impairment levels.

## Background

Multiple sclerosis (MS) is a neurologic disease affecting an estimated 2.5 million adults worldwide and is the most common disabling neurological disease in young adults [1]. Central nervous system damage associated with the disease often results in ambulatory limitations, key components of disability in people with MS (PwMS) [2]. Approximately 80% of these patients experience clinically significant walking difficulties [3] presenting even in the early stages of the disease and in individuals diagnosed with clinically isolated syndrome [4]. Moreover, the progressive loss of ambulation results in compromised participation of social engagement, unemployment and a poorer quality of life [5].

To date, there is no curative treatment for MS. Despite the fact that drug-induced immunosuppression and immunomodulation have been shown to decelerate the inflammatory-related progression of MS [6], extensive disabilities such as a decline in mobility occur during the course of the disease and require specific symptomatic treatments. Symptomatic treatments involve not only drugs but a substantial number of functional interventions, specifically physical therapy techniques, an essential part of comprehensive rehabilitation programs.

Numerous clinical trials have examined the effect of physical therapy rehabilitation programs on ambulatory outcomes in PwMS [7-19]. Walking speed improves following combined or isolated aerobic or resistance training [12-15] as well as after stability [16] or aquatic training [17]. Progress in walking endurance are primarily seen following aerobic training [7,10,18,19] though, resistance training [15] or combined aerobic and resistance training exercises [12] are also beneficial. As quantified in several meta-analysis reviews, the evidence corroborates positive effects of physical and exercise training on walking speed and endurance in PwMS [20,21].

For the past 10 years, the Sheba MS Center inaugurated an intense 3-week rehabilitation program specifically designed for MS patients with various neurological impairments. The primary goal of the program is to improve physical capabilities thus enhancing functional and daily activities. The original idea was drawn from Romberg et al’s study in 2004 who reported improvements in short and long gait measures following a 6-month physical rehabilitation program performed on PwMS [12]. Romberg’s program consisted of strength and aerobic training carried out in two different locations and an intense 3 week inpatient period followed by a 23 week program at home. The Sheba MS Center rehabilitation program is consists of a 3 week inpatient period with the exception that all physical activities are goal-directed and personalized according to the participant’s specific needs.

A particular limitation of Romberg’s study was associated with their population group. The MS participants exhibited varied neurological impairments, expressed by the relatively large range of the Expanded Disability Status Scale (EDSS) score (1.0-5.5), preventing the ability to determine whether the improvements in walking were similar for all disability levels. Worth noting, this drawback has been observed in other trials examining different intervention options directed towards improving mobility in the MS population. Furthermore, it is crucial to examine physical and exercise training modalities and outcomes, especially in people with advanced MS, given that disease-modifying therapies have become less effective in advanced stages of MS [22].

Therefore, the primary objective of the current study was to report on the effectiveness of a personalized, intense tailored, 3 week physical rehabilitation program, on walking in a large group of PwMS. Furthermore, we examined whether gait outcomes were dependent on the level of neurological disability.

## Methods

### Study population and data extraction

We accessed the Sheba MS’s computerized database, a population-based registry documenting demographic and clinical data of all MS patients followed at the Sheba Medical Center, Tel-Hashomer, Israel from January 1, 1995. The MS Center provides long-term multidisciplinary care and treatment for patients from referral areas all over the country diagnosed with MS. The Center is currently following and treating 3250 patients out of ~5000 PwMS.

Since the establishment of the MS Center, an electronic record-keeping system has documented the patients’ demographic, clinical and imaging data. This system is updated by the Center’s medical staff during each visit to the clinic. Every 6 months a complete neurological examination is performed and an EDSS score assigned and recorded. The EDSS, an accepted method of quantifying disability in PwMS consists of an eight-function system scale monitoring motor, sensory, cerebellar, brain stem, visual, bowel and bladder, pyramidal and other functions. Each domain is graded from 0 = no disability to 5 or 6 = maximal disability [23]. According to the score achieved from each functional system, an integrated score between 0 = normal examination and 10 = death from MS is derived. A score ranging from 1.0 to 4.5 denotes patients who are fully ambulatory without aid; a score of 5.0 to 7.5 reveals moderate to severe impairment in ambulation and a score of 8.0 to 9.5 refers to PwMS essentially restricted to bed.

To ensure our goal, we extracted data of all MS patients who had participated in the center’s physical rehabilitation program. Patients suffering from orthopedic and/or cardiovascular pathologies negatively affecting walking were excluded. The program’s basic structure, maintained since its initial launch, consists of 36 one-on-one treatment sessions spread over 3 consecutive weeks. Every treatment session, including a brief description of its content is recorded daily. In order to preserve homogeneity of the included participants, we extracted only those patients who received at least 30 treatment sessions throughout the rehabilitation period. The integrity of the data registry was evaluated by a computerized logic-algorithm-questioning process identifying data entry errors. The study was approved by the Sheba Institutional Review Board. All participating subjects signed an informed consent form for use of their data in the research projects.

### Structure and components of the physical rehabilitation program

Approximately one week prior to initiation of the program, every patient was requested to appear at the MS Center for assessment and consultation. The evaluation session was executed by a neurologist and physical therapist; both specialized in MS and considered the case managers of the specific patient during the program. The main purpose of the initial meeting is to determine the patient’s major physical limitations. Furthermore, the medical team, in sync with the patient, defines the major functional goals of the upcoming program. Prior to initiation of the program, information is documented and furnished to the therapists involved in the program.

According to the defined main goals, specific targets were implemented by: (a) goal directed physical therapy (a 45 minute session, 5 times a week) aimed at increasing muscle strength, improving balance and gait, decreasing spasticity and improving functional activities of daily living; (b) moderately intense aerobic exercise training on a bicycle ergometer (a 45 minute session, 3 times a week). The aerobic exercise intervention is designed to improve cardiorespiratory fitness with an exercise intensity prescription derived from peak heart rate (HR) responses to baseline graded exercise testing. Intensity levels and exertion were monitored by trained exercise practitioners; (c) aquatic therapy (a 45 minute session, twice weekly) chiefly oriented to body structures appropriate to movement. Therapy domains mainly focused on trunk mobility exercises, postural stability, transferring oneself and changing body positions. In addition to the physical components of the program, each patient met several times with a nurse, psychologist, social worker and occupational therapist. The aim of these single consultation sessions were to ensure that the overall needs of the PwMS were being addressed. In the event where an additional specific medical domain was required, meetings were scheduled after termination of the 3 week rehabilitation program. Worth noting, data gathered by these health professionals were not quantified, thus they were not included in the statistical analysis.

Importantly, every week the rehabilitation staff of the MS Center conducted a general meeting where the case managers reported progress or difficulties of the participating patients. Furthermore, at the end of the final week, the patient was invited to the meeting to share and summarize his thoughts regarding the rehabilitation period.

Protocols of all sessions were stored in the computerized data system. Protocols included information regarding the medical personnel responsible for the session and a free text box. The free text option allowed the clinician to document various issues relating to the patient’s performance and/or a session that in his view was essential for follow up (e.g motivation, mood, fatigue during practice, etc). Unfortunately, this data was not organized in a form that allowed statistical analysis.

It is worth noting, despite changes in personnel throughout the last decade, the process detailed in the previous section has been consistently maintained with minimal variations.

#### Gait measurements

The MS Center evaluates walking abilities via different clinical ambulation and balance tests (e.g. 6-minute walk test, Timed 25-Foot Walk, Functional Reach Test, etc). We extracted gait tests that have been continuously used since the inception of the rehabilitation program. The referred walking tests were assessed at baseline and at the end of the 3-week rehabilitation program.

### Long test, 2-minute walk test (2mWT)

Subjects were instructed to complete the test ‘at their fastest speed and cover as much distance as possible by walking back and forth down a 30 meter hallway, circling cones at each end and notified after each expired minute. They were allowed to use their habitual assistive device. Total distance was then registered. Walking improvement on the 2mWT was indicated by positive score changes (in meters). Recently reported, the clinical meaningful change in the 2mWT from the MS patient’s perspective was defined as 9.6 m [24].

### Short tests, the 10–20 meter walking time (10mwt, 20mwt)

Subjects were instructed ‘to walk safely at the fastest speed’. A dynamic start was adopted and time measurement occurred over the midpoint 10–20 meters of a 30 meter walkway. Measurement did not include initial and terminal steps in order to avoid measuring the acceleration and deceleration phases of gait. The use of an assistive device such as a foot orthosis or cane was recorded; the same walking device was used at each session. Walking improvement on the short walking tests was reflected by negative score changes in seconds. According to previous studies, a genuine change in short walking tests in PwMS ranges from 23% up to 38% [25,26].

### Timed up and go test (TUG)

The TUG requires both static and dynamic balance. The individual may wear their usual footwear and can use any assistive device they normally use. The starting point is determined when the subject is seated in a chair with their back flush against the chair and their arms resting on the arm rests. He/she is then instructed by the examiner to stand up, walk 3 meters, turn around, walk back to the chair and sit down again. Timing begins when the individual begins to rise and ends when he/she returns to the chair and sits down. Walking improvement on the TUG is indicated by negative score changes (in seconds). Learmonth et al. [27] reported that a threshold of a 10.6 s decrease in the TUG was correlated with a meaningful change in walking performance in PwMS.

### Statistical analysis

Descriptive statistics were used to characterize the population. Patients were subdivided into three levels of disability based on their EDSS score. Patients with an EDSS score <4.5 were considered as mildly disabled; those who scored 4.5 up to 5.5 were considered moderately disabled and those with scores of 6.0 and 6.5 (indicating assistance with walking aids) were considered severely disabled. The Kolmogorov-Smirnov goodness of fitness test for independent samples tested for normality of the distribution of all parameters. For all parameters, the significance level was <0.05, indicating a normal distribution. Thus, differences in gait measurements from baseline to termination of the 3 week physical rehabilitation program were determined by the repeated measures ANOVA tests. The post-hoc Bonferroni test enabled paired multiple comparisons between groups. Changes in gait performance were calculated in percentages (%). Negative scores in the 10-20 m and TUG walking tests as well as positive scores in the 2mWT signified an improvement in ambulation abilities. The magnitudes of differences were indexed by a 95% confidence interval (95% CI).

In order to identify predictors related to improvement in the long walking distance test, a stepwise multivariate linear regression analysis was performed. The pre-post change in the 2MWT was defined as the dependent variable while gender, age, disease duration and EDSS (with subcategories) were defined as explanatory variables. All analyses were performed using IBM SPSS statistics software (Version 22.0 for Windows, SPSS Inc. NY, USA). All reported P-values were two-tailed. A nominal level of 0.05 was used for all comparisons.

## Results

Of the 3,250 patients registered in the Sheba MS’s computerized database, 2,762 were classified with relapsing-remitting MS according to the revised McDonald criteria [28]. From this group, a total of 381 participated in the 3 week rehabilitation program. Sixty-nine patients were not assessed after the rehabilitation period due to unforeseen medical problems (ie, flu, fracture) or an error in the administration process (ie results entered incorrectly into the medical database or accidently not assessed at the final intervention session). Nevertheless, this group did not significantly differ from the group included in the analysis (n = 312) as to age, gender, EDSS and duration of disease. The total group was classified according to level of ambulation disability derived from the EDSS score. Categorization included mild (EDSS < 4.5, n = 87), moderate (EDSS = 4.5-5.5, n = 104) and severely (EDSS = 6.0-6.5, n = 121) disabled. Characteristics of the participants in the 3 week rehabilitation program are provided in Table 1.

**Table 1:** **Demographic and clinical characteristics of the MS study group**

**Variable**	**Mean (S.E)**			
	**Entire group (n = 312)**	**Mild disability (EDSS range <4.5) (n = 87)**	**Moderate disability (EDSS range 4.5-5.5) (n = 104)**	**Severe disability (EDSS 6.0-6.5) (n = 121)**
Age (years)	44.6 (0.7)	42.4 (1.3)	43.0 (1.1)	47.7 (1.0)
Gender				
Female (n, %)	205, 65.7%	56, 64.4%	72, 69.2%	77, 63.6%
Male (n, %)	107, 34.3%	31, 35.6%	32, 30.8%	44, 36.4%
Disease duration (years)	9.0 (0.43)	7.3 (0.77)	8.0 (0.60)	11.2 (0.76)
EDSS	5.0 (0.06)	3.6 (0.07)	5.0 (0.04)	6.1 (0.02)
Pyramidal	3.2 (0.14)	2.6 (0.09)	3.0 (0.06)	3.9 (0.33)
Cerebellar	1.8 (0.07)	1.5 (0.13)	1.8 (0.11)	2.2 (0.12)
Sensory	1.5 (0.07)	1.2 (0.12)	1.5 (0.59)	1.7 (0.11)
Cerebral	0.3 (0.12)	0.2 (0.14)	0.3 (0.11)	0.3 (0.13)

EDSS -expanded disability status scale.

**Figure 1:**
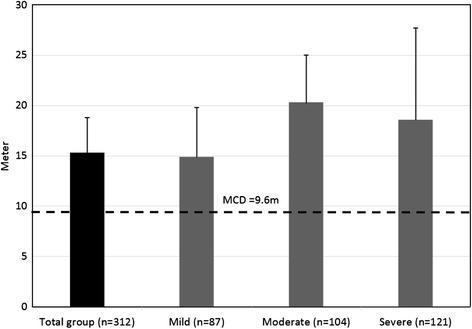
**Pre-post rehabilitation program mean difference in the 2mWT.** MCD, Minimal Clinical Difference, based on (Baret et al, 2014).

Pre and post-intervention gait outcome measures, according to MS disability groups, are presented in Table 2. In terms of the 2mWT, a significant improvement was observed in all three MS subgroups. Increase in walking distance (in meters) in the mild, moderate and severe MS groups was 14.9, 20.3 and 18.6, respectively. Importantly, the improvements in all MS groups were above the minimal clinical difference of 9.6 m defined by Baret et al’s study of PwMS [24] (Figure 1). Post-hoc comparisons did not reveal differences in the mean amount of improvement between any two disability groups (*P*-value >0.05).

**Table 2:** **Changes in gait measurements following a physical rehabilitation program**

**Variable**	**Mean (S.E)**				
	**Pre**	**Post**	**Mean difference (95% CI)**	**P- Value**	**Pre-post change (%)**
**Entire group** (EDSS range 2–6.5) (n = 312)					
10mWT (s)	15.4 (0.7)	13.0 (0.6)	−2.3 (−1.4, −3.3)	<0.001*	−10.1 (1.7)
20mWT (s)	33.8 (1.7)	29.0 (1.6)	−4.8 (−2.7, −6.9)	<0.001*	−10.6 (1.7)
2mWT (m)	120.8 (3.1)	136.1 (3.8)	15.3 (11.2, 19.5)	<0.001*	14.9 (1.9)
TUG (s)	15.8 (0.8)	14.2 (0.6)	−1.6 (−0.7, −2.6)	0.001*	−5.4 (1.6)
**Mild disability** (EDSS range <4.5) (n = 87)					
10mWT (s)	9.5 (0.5)	8.4 (0.4)	−1.1 (−1.7, −0.6)	<0.001*	−9.2 (2.3)
20mWT (s)	20.1 (1.0)	17.9 (0.8)	−2.1 (−0.9, −3.4)	0.001*	−7.7 (2.3)
2mWT (m)	143.2 (4.3)	158.1 (5.6)	14.9 (8.7, 21.1)	0.001*	10.9 (2.3)
TUG (s)	9.8 (0.5)	8.9 (0.3)	−0.9 (−1.5, −0.3)	0.006*	−5.1 (2.4)
**Moderate disability** (EDSS = 4.5-5.5) (n = 104)					
10mWT (s)	11.5 (0.5)	9.9 (0.4)	−1.6 (−1.0, −2.2)	<0.001*	−10.3 (2.1)
20mWT (s)	24.5 (1.0)	21.2 (1.1)	−3.4 (−2.1, −4.7)	<0.001*	−11.3 (1.9)
2mWT (m)	120.3 (4.2)	140.6 (5.2)	20.3 (13.1, 27.5)	<0.001*	19.0 (3.4)
TUG (s)	12.3 (1.1)	10.8 (0.4)	−1.5 (−0.6, 3.5)	0.156	−3.9 (2.3)
**Severe disability** (EDSS 6.0-6.5) (n = 121)					
10mWT (s)	23.2 (1.5)	19.2 (1.4)	−4.0 (−6.4, −1.6)	0.001*	−10.5 (3.9)
20mWT (s)	52.3 (3.6)	43.1 (3.7)	−9.2 (−14.1, −4.2)	<0.001*	−12.1 (3.7)
2mWT (m)	94.5 (7.4)	113.1 (11.2)	18.6 (6.7, 30.6)	0.004*	16.2 (5.4)
TUG (s)	23.8 (1.6)	21.4 (1.4)	−2.4 (−4.2, −0.6)	0.011*	−6.8 (3.1)

EDSS- expanded disability status scale; 10mWT- ten-meter walking test; 20mWT- twenty-meter walking test; 2mWT- two-minute walking test; TUG - timed up and go test. *P<0.05.

Regarding the short walking tests (10mWT and 20mWT); improvements were demonstrated in all disability groups. However, the improvement (represented by a higher velocity or a decrease in total time period to complete the defined distance) was relatively small. The mean reduction in total time for the entire group was 2.3 and 4.8 (sec) in the 10mWT and 20mWT, respectively. In terms of % pre-post change, improvements in both tests ranged from 7.7 to 12.1. These values are below the minimal clinical difference threshold presented by previous reports [25,26].

**Figure 2:**
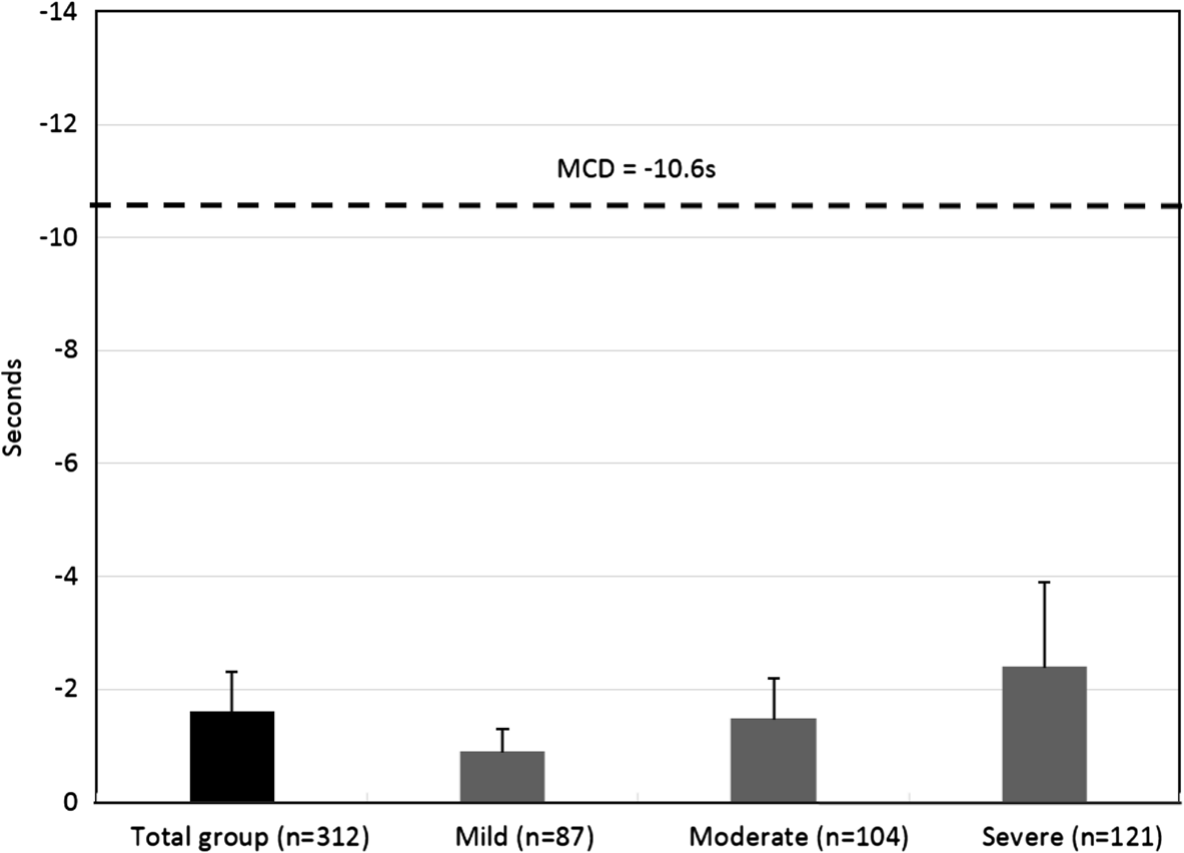
**Pre-post rehabilitation program mean difference in the timed up and go test.** MCD, Minimal Clinical Difference based on (Learmonth et al, 2012).

Results of the TUG were similar to those of the short walking tests. Significant improvements were demonstrated in the mild (0.9 sec) and severe (2.4 sec) groups. Non-significant changes were found in the moderate group. However, improvements in the mild and moderate groups were significantly below the minimal clinical difference determined in previous reports [27]. TUG results are presented in Figure 2.

Regarding the pre-post % change, the improvement in the 2mWT was significantly greater compared to the short walking and TUG [F(3, 624) = 91.0, *P*-Value < 0.001]. Additionally, participants in the moderate and severe groups improved to a larger extent compared to the mild group. Mean % pre post change on the 2mWT were 19.0 (S.E. = 3.4), 16.2 (S.E. = 5.4) and 10.9 (S.E. = 2.3), respectively (Figure 1). In the same context, the % pre post change in the TUG was significantly lower compared to the % of change related to the long and short walking tests [F(3, 624) = 53.0, *P*-Value < 0.001].

According to the multivariate linear regression model, the EDSS score and disease duration were the only explanatory parameters found to be significant variables (β = 0.253, R^2^ = 0.072, F_(1, 67)_ = 6.606, P = 0.002). Gender, age and EDSS subcategory variables were excluded from the model. On the basis of this analysis, the general neurological impairment level score and disease duration of the PwMS accounted for 7.2% of the variance of the pre-post improvement in the 2MWT.

## Discussion

The aim of this retrospective study was to examine the effectiveness of a 3 week goal-directed comprehensive physical rehabilitation program on walking abilities in PwMS. The relatively large number of participants included in the final analysis (n = 312) were classified according to level of disability (mild/moderate/severe) based on their EDSS scores. Our results demonstrated a significant positive effect of the intense physical rehabilitation program on walking performance in PwMS, notwithstanding level of disability. The key improvement was demonstrated in terms of the 2mWT outcome, exhibiting a mean increase of 15.3 m (mean 14.9% pre-post change).

However, to examine whether changes in gait performance were clinically meaningful, we compared our results with meaningful clinical change reference values in walking tests performed in Pw MS [24-27].

Nilsagard et al. [25] reported that for the 10mWT and the TUG, a 23-24% improvement establishes a genuine change. For the 30mWT, 14% was determined for MS patients suffering from mild neurological impairments (EDSS ≤4) and 38% in those suffering from moderate neurological impairments (EDSS >4). Hobart et al. [26] reported that a 20% reduction of the Time to Walk 25-feet test should be used as the threshold value that reliably indicates a true change in walking for an PwMS.

Furthermore, Learmonth et al. [27] stated that a threshold of a 10.6 s decrease in the TUG is correlated with a meaningful change in walking performance in PwMS with moderate neurological impairment (EDSS 5–6.5). Recently, the Rehabilitation in MS (RIMS) Mobility Research Group found that in the 2mWT, the clinical meaningful change from the patient’s and therapist’s perspective was 9.6 m and 6.8 m, respectively [24].

Although our findings confirm a significant positive change in the 10mWT, 20mWT measures following the rehabilitation program, the percentage of change (ranging from 7 to 12) was clearly below the minimal detectable change values suggested by previous reports [25-27]. This observation is valid regarding the TUG. In the present study, TUG findings ranged from 3.9 to 6.8 percentage of change.

In contrast, findings related to the 2mWT (ranging from 14.9 m to 20.3 m) exceeded the minimal detectable change values presented by Baret et al. [24]. Worth noting, these authors stated that the 2mWT is one of the most appropriate walking measures for evaluating the effect of physical rehabilitation interventions in PwMS.

We believe that the effectiveness of the rehabilitation program on ambulation was obscured by the short walking tests. This claim is reinforced by different studies evaluating gait measurements in PwMS, reporting that longer walking tests should be preferred over short walking measurements in assessing walking fatigability, distance limitations and functional capacity [29]. In the same context, there is a chance that due to a floor and ceiling effect of the short walking tests, the improvements in terms of walking distance and speed were not captured in the study population, especially in the mild and moderate MS subgroups.

An important strong point of this study is the relatively large sample size allowing analysis of specified patient groups, separated according to level of walking disability. Accordingly, in the current study, percentage of change in the 2mWT was higher in the moderate and severely gait groups compared to the mild group participants, 19.0 (S.E. = 3.4), 16.2 (S.E. = 5.4), 10.9 (S.E. = 2.3), respectively. Additionally, according to the multivariate regression model, a higher score on the EDSS scale was associated with a greater improvement on the 2MWT. These significant observations are in line with previous trials examining the effects of physical rehabilitation programs on muscle strength and ambulation in PwMS with moderate-severe neurological impairments [30,31].

Filipi et al’s study (2011) of 67 PwMS, created a 6 month muscle resistance program demonstrating ignificant improvements in strength and endurance in individuals with moderate neurological levels (EDSS 5–7.5) [30]. Similarly, following a web-based 6 month physical activity intervention program, improvements in walking distance (according to the 6 min walking test) were equally effective in persons with mild and moderate disabilities [31]. Furthermore, these observations confirm the conclusions of a large meta-analysis stating that among PwMS with moderate MS disabilities, sufficient evidence exists that exercise training is effective in improving aerobic capacity, muscular strength, mobility, fatigue, and health-related quality of life [32].

In contrast, other studies have reported greater effects of exercise training on walking mobility outcomes in mildly disabled PwMS compared with the moderately disabled [33]. Hence, additional trials comparing the effects of physical rehabilitation programs on walking abilities based on disability level are still essential.

Importantly, ambulation improvements documented in the present report were achieved after a relatively short period. Compared with previous trials implementing physical rehabilitation programs in the MS population [7-21], our study was the shortest. On the other hand, the frequency of practice sessions was intense.

We believe that our study abolishes a historical belief that it is not prudent to often train MS patients due to fatigue effects and that walking improvements in PwMS can be safely achieved in a realistic manner, in just 3 weeks.

The current study has several limitations. Firstly, the study design is a retrospective cohort relying on accurate record-keeping. Several cases may have been missed and/or inaccurately recorded. In order to avoid this, the final database files were individually double-checked by an expert in bioinformatics. Secondly, data regarding the follow up period was not provided. Indeed, future studies should explore the effects of long-term physical rehabilitation programs with a special interest on the effect of disease progression.

Another limitation was the absence of an active control group. No clear confirmation was presented as to whether changes were due to increased physical activity rather than a time effect. Additionally, due to the combined nature of the physical intervention program, we could not differentiate between the specific session contents. Nevertheless, our aim was to evaluate the program as a whole and examine its effects in different MS patient groups. Finally, data analysis included only 4 gait measurements. If additional outcome measurements such as the patient-reported Multiple Sclerosis Walking Scale-12 or the 6-minute Walk Test, fatigue and level of spasticity, would have been assessed, additional conclusions could have been drawn.

## Conclusions

The current study provides valuable data relating to the positive effects of a tailored, integrated physical rehabilitation program on ambulation in the MS population. Moreover, walking improvement was achieved despite level of disability. Importantly, meaningful clinical walking improvement was reflected solely in terms of a long distance walking test.

Our primary observations are helpful for all medical practitioners involved in physical rehabilitation programs for PwMS. The present information should encourage centers that are currently providing rehabilitation programs directed to the MS population. In addition, this research may inspire additional MS Centers to follow the protocol. Nevertheless, future studies should investigate whether the increase in walking distance and gait velocity after training is correlated to augmenting brain synaptic plasticity. Furthermore, an effort should be made to define a dose–response relationship between definite physical training components (intensity, duration, type, etc.) and specific functional disabilities (balance, gait, fatigue, spasticity, etc.) of the MS population.
